# A randomized controlled trial to investigate the influence of low dose radiotherapy on immune stimulatory effects in liver metastases of colorectal cancer

**DOI:** 10.1186/1471-2407-11-419

**Published:** 2011-09-30

**Authors:** Christoph Reissfelder, Carmen Timke, Hubertus Schmitz-Winnenthal, Nuh N Rahbari, Moritz Koch, Felix Klug, Falk Roeder, Lutz Edler, Jürgen Debus, Markus W Büchler, Philipp Beckhove, Peter E Huber, Jürgen Weitz

**Affiliations:** 1 Department of General, Visceral and Transplantation Surgery, University Hospital Heidelberg, Germany; 2 Department of Radiation Oncology, German Cancer Research Center and University Hospital Center, Heidelberg, Germany; 3 Translational Immunology Unit, German Cancer Research Center, Heidelberg, Germany

**Keywords:** colorectal liver metastasis, low dose radiation, tumor specific T cells

## Abstract

**Background:**

Insufficient migration and activation of tumor specific effector T cells in the tumor is one of the main reasons for inadequate host anti-tumor immune response. External radiation seems to induce inflammation and activate the immune response. This phase I/II clinical trial aims to evaluate whether low dose single fraction radiotherapy can improve T cell associated antitumor immune response in patients with colorectal liver metastases.

**Methods/Design:**

This is an investigator-initiated, prospective randomised, 4-armed, controlled Phase I/II trial. Patients undergoing elective hepatic resection due to colorectal cancer liver metastasis will be enrolled in the study. Patients will receive 0 Gy, 0.5 Gy, 2 Gy or 5 Gy radiation targeted to their liver metastasis. Radiation will be applied by external beam radiotherapy using a 6 MV linear accelerator (Linac) with intensity modulated radiotherapy (IMRT) technique two days prior to surgical resection. All patients admitted to the Department of General-, Visceral-, and Transplantion Surgery, University of Heidelberg for elective hepatic resection are consecutively screened for eligibility into this trial, and written informed consent is obtained before inclusion. The primary objective is to assess the effect of active local external beam radiation dose on, tumor infiltrating T cells as a surrogate parameter for antitumor activity. Secondary objectives include radiogenic treatment toxicity, postoperative morbidity and mortality, local tumor control and recurrence patterns, survival and quality of life. Furthermore, frequencies of systemic tumor reactive T cells in blood and bone marrow will be correlated with clinical outcome.

**Discussion:**

This is a randomized controlled patient blinded trial to assess the safety and efficiency of low dose radiotherapy on metastasis infiltrating T cells and thus potentially enhance the antitumor immune response.

**Trial registration:**

ClinicalTrials.gov: NCT01191632

## Background

Colorectal cancer (CRC) is the most frequent cancer in the western world [1]. Advanced tumor stage with metastasis is one of the main reasons for the high mortality rate. At the time of diagnosis, approximately 30% of patients have distant metastases which predominantly occur in the liver. Surgical removal of the tumor still remains the only curative approach [2,3]. In recent years survival rates in colorectal cancer have been further improved because of multimodal therapy concepts, especially in UICC stage III and IV patients. In parallel to these modern multimodal therapy concepts, novel and promising concepts including immunotherapeutic strategies are actively being investigated to further improve clinical outcome.

Most patients with CRC have high numbers of tumor specific T cells (cytotoxic T cells and T helper cells) in their blood and bone marrow [4,5]. The molecular identification of tumor-associated antigens (TAA) has led to the development of antigen-specific immunotherapy targeting these antigens. To the present these strategies have failed to show efficacy in clinical practice. Potential reasons for this lack of efficacy include the inefficiency of T cell activation in vivo, insufficient migration of cytotoxic T cells into the tumor, the choice of irrelevant antigens, induction or application of monovalent T cells or the treatment of patients in highly advanced tumor stages [6]. In particular inadequate T cell infiltration, characterized by selective recruitment of immune suppressive regulatory T cells as well as insufficient immigration of cytotoxic T cells [7-9] appears to be a serious hurdle, partially explainable by poorly activated endothelium of the tumor vessels [10]. Furthermore, the cytotoxic and secretory activity of tumor specific T cells may be inhibited by the immunosuppressive environment of the tumor stroma after immigration into the tumor.

Ionizing radiation therapy (RT) is an effective local cancer therapy, which kills cancer and other cells within the tumor stroma, including endothelial cells and intratumoral lymphocytes [11]. RT typically induces primarily mitotic cell death but also leads to apoptosis [12]. Radiation has also complex effects on the tumor microenvironment and microvessels, which can facilitate the homing of both antigen presenting and effector T cells to the tumor [10,13].

Tumor cells killed by RT may be a good source of antigens for Dendritic Cell (DC) uptake and presentation to T cells. DCs are attracted by the altered microenvironment and undergo maturation after internalizing the debris. These dendritic cells can then present tumor specific antigens to T-cells following the processing of debris [14]. The possibility of using RT to promote tumor antigen-presentation by DC has been explored. Sublethal doses of ionising radiation have been described to enhance antitumor T-cell activity in such indirect ways in animal studies. Radiation has also been described to directly lead to an amplification of expression of MHC. This can render tumor cells more sensitive to the detection and lysis by specific T-cells [15]. Furthermore ionizing radiation can lead to activated endothelium thus facilitating the immigration of T-cells into the tumor. Overall, the available preclinical in vivo data support the hypothesis that RT may provide at least some of the necessary maturation signals.

So far, no systematic clinical analysis has been performed to investigate the immune stimulatory effects of low dose radiotherapy in patients with CRC. One issue to be addressed is the effective radiation dose to act as a form of immunotherapy. In fact, scant data are available with regard to the specific radiosensitivity of the different cellular components of the immune system. Studies describing the effects of RT on circulating immune cells are also lacking along with data on radiation effects on the microenvironment.

We here present the protocol of a clinical phase I/II trial with the aim to determine the active local external beam radiation dose resulting in tumor infiltrating T cells in patients with liver metastasis from CRC. The liver metastases will be irradiated two days prior to surgery using external beam radiotherapy. As primary endpoints we will determine the active local radiation dose leading to metastasis infiltrating T cells as a surrogate parameter for antitumor activity. Secondary objectives include local tumor control, survival, treatment toxicity as well as quality of life. Furthermore, blood cell transcriptomics and plasma-proteomics will be correlated with the amount of tumor reactive T cells in blood and bone marrow.

## Methods/Design

### Trial population and patient recruitment

This trial focuses on patients with resectable liver metastases of CRC. Patients scheduled for elective hepatic resection due to colorectal liver metastasis will be enrolled in this study. Only patients over 50 years of age are allowed to participate in this study due to the radiation protection law. Based on the preoperative imaging the surgeon determines if the liver metastasis can be resected in curative intent. Patients with unresectable liver metastasis, secondary malignancies, liver cirrhosis or previous radiotherapy to the upper abdomen will be excluded from the trial (see Table 1).

**Table 1:** Eligibility criteria

Inclusion criteria
Liver metastasis of colorectal cancer
Metastasis is curatively resectable
Written informed consent of the patient
No evidence of active or former concurrent malignant diseases

**Exclusion criteria**

Previous radiotherapy to the upper abdomen
Participation in other therapeutical trials
Liver cirrhosis
Pregnancy
No willingness on regular follow up care
Any extrahepatic disease

Patients who meet the eligibility criteria and provide written informed consent are randomly allocated to either study intervention to balance treatment groups for all known and unknown potentially confounding factors. A block randomisation list is created via a computer system. Patients are randomised using consecutively numbered opaque envelopes prepared and sealed by an independent study nurse. The envelopes will be opened sequentially only after the participant's name is written on the appropriate envelope. Neither the screening investigators nor the investigators who inform the potential participants about this trial and enrolling the patients, have any knowledge of the block randomisation list or the previous allocations to treatment. There is no stratification for randomisation.

After informed consent, patients are randomized either to the control group - receiving no radiation - or the three irradiation treatment groups. Within the irradiation treatment groups patients will receive 0.5 Gy, 2 Gy or 5 Gy single fraction radiation 48 h prior to resection. After the adjustment of an individual positioning device, computed tomography and, if needed, magnetic resonance imaging (MRI) treatment planning examinations are performed. After 3D inverse treatment planning and plan optimizing using VIRTUOS and KONRAD software, intensity modulated radiation therapy will be administered using five to nine 6 MV linear acceleration (LINAC) photon beams. Immediately prior to the irradiation the setup error of the patient will be determined via In-Room-CT, and corrected if necessary.

The first blood sample will be obtained (54 ml) before radiation, a second just prior to the start of the operation. A bilateral bone marrow sample (27 ml each) will be taken from both iliac crests after induction of general anaesthesia just before skin incision. Bone marrow sampling is optional for the patient. EDTA will be used as anticoagulant for bone marrow and blood samples. After hepatic resection a part of the tumor will be immediately removed and stored at -80°C for further analyses.

On the postoperative day seven and at the follow-up/restaging visits (4 weeks postoperatively, four and seven months postoperatively) additional venous blood samples (54 ml) will be obtained. Quality of Life will be tested using the EORTC QLQ C-30 at four months and seven months postoperatively. Clinical examination will be performed and blood samples will be obtained by the investigators at all visits.

Curative surgery requires complete resection of all liver metastases, regardless of size, number, distribution, or width of resection margin. Liver operation will be carried out under low central venous pressure using a Péan clamp, ultrasonic dissector or vascular stapler. An intraoperative ultrasound of the liver will be performed in all patients to assess the relationship of the lesions to major vessels and to rule out additional lesions. The Brisbane 2000 terminology of hepatic anatomy and resections will be applied [16].

### Safety aspects and adverse events

As the radiation dose is relatively low, very few side effects are expected. Potential adverse events (AE) include skin alterations like redness, temporary elevated transaminases, transient nausea or diarrhea. All possible AEs will be assessed by the RTOG Scale. These AEs rarely occur in conventional radiotherapy using considerably higher doses (neoadjuvant 5 × 5 Gy for rectal or gastric cancer, 54 Gy in pancreatic cancer) [17,18].

### Study design

This single fraction radiation on colorectal liver metastases trial is a registered (ClinicalTrials.gov - NCT01191632), investigator-initiated, prospective, randomized controlled phase I/II trial to evaluate the optimal neoadjuvant, single fraction radiation dose for patients undergoing resection for colorectal liver metastases in curative intent. Patients will be randomised either to the control group -receiving no radiation- or the three treatment groups - receiving 0.5 Gy, 2 Gy or 5 Gy single fraction radiation to the metastasis 48 h prior to the operation. The treatment is offered to a heterogeneous group of patient of both sexes, covering a wide range of comorbidities. For the irradiation a highly conformal dose distribution to the tumor with 1 cm safety margin is delivered using a 6 MV LINAC and intensity modulated radiotherapy (IMRT) treatment application. After inclusion of 20 patients an interims analysis will be performed. The final analysis will be performed within three month of the inclusion of the last patient. The trial design will not be changed without prior agreement of the ethics committee.

Translational studies will be carried out by the Department of Translational Immunology of dkfz, the Radiation Oncology Departments of German Cancer Research Center (DKFZ) and the Surgery Departments of the University Hospital. This trial is an investigator-initiated trial and is coordinated by the Department of General-, Visceral- and Transplantation Surgery and the Radiation Oncology Department of DKFZ. The Radiation Oncology Department of DKFZ is responsible for the overall trial management, trial registration, database management, quality assurance including monitoring and scientific program of all trial related meetings. The methods and the study design of this trial have the same structure as the previously published study protocol for patients with pancreatic cancer (BMC Cancer 2011; 11:34) [19].

### Ethical and legal considerations

The final clinical trial protocol, the patient information and informed consent sheets were approved by the independent ethics committee of the University of Heidelberg S081/2008 (http://klinikum.uni-heidelberg.de). Furthermore, the study has been approved by the German Federal Authorities for Radiation Protection (Bundesamt fuer Strahlenschutz, Salzgitter, Trial Number Z5-22461/2-2009-002). Written informed consent is obtained from each patient in oral and written form before inclusion in the study. In addition, the protocol has been reviewed and funded by a scientific third party funding (grants from Nationales Centrum fuer Tumorerkrankungen (NCT)/Tumorzentrum Heidelberg and Kompetenzverbund Strahlenforschung (KVSF, 03NUK004A,C) of Bundesministerien fuer Bildung, Forschung und Umwelt (BMBF/BMU)). Patients are informed about the strict confidentiality of their personal data within this trial, but their pseudonymised medical records may be reviewed for trial purposes by authorized individuals other than their treating physician.

It has to be pointed out that the participation is voluntary and that the patient is allowed to refuse further participation in the protocol to any time point within the study. This trial is carried out in accordance to the current Declaration of Helsinki (sixth revision, 2008), the principles of "Good Clinical Practice" (GCP), and the Federal Data Protection Act. It is registered at the Clinical-Trials.gov protocol registration system (http://www.clinicaltrials.gov) and the assigned identification number is NCT01191632.

### Objective of the study

The primary objective of this trial is the determination of the single fraction radiation dose, measured by the number of tumor infiltrating T-cells in the tumor. Secondary molecular objectives are the T cell activity in the resected tumor specimen, the density of regulatory T cells, and the frequency of tumor reactive T cells in blood and bone marrow in correlation with changes in the level of proteins involved in immune response or angiogenesis and transcriptomics in whole blood cells. Further clinical secondary objectives are acute radiogenic toxicity, postoperative morbidity and mortality, local control, recurrence patterns, overall survival and quality of life. All clinical secondary objectives are presented in Table 2.

**Table 2:** Secondary endpoints

Secondary endpoints
• Local control and recurrence patterns of colorectal liver metastasis • Progression free survival • Surgical morbidity in patients undergoing liver resection who received this protocol treatment • 30 day postoperative mortality • Radiation toxicity • Quality of life according to EORTC QoL questionnaire after 3 and 6 months

Currently, 35 patients are randomized. The duration of the trial is expected to last four more months. Usually, two patients per month can be enrolled into this study. Evaluation and reporting of the clinical and laboratory results will be done within three months after the end of recruitment and closing of the database.

### Statistical analysis

This randomized controlled trial is planned as a comparison of four parallel groups:

Group 1: Dose = 0 Gy (control); Group 2: Dose = 0.5 Gy; Group 3: Dose = 2 Gy and Group 4: Dose = 5 Gy.

Aim of the study is to determine the maximum active radiation dose with three different doses. This will be carried out hierarchically in two consecutive steps.

1. Step: Comparison of the three doses against the control group ("Many-to-one" comparison after Dunnet)

2. Step: Comparison of the three doses among each other ("all-subset" comparison)

Each of the two steps happens on multiple levels of significance with a p-value less than 0.05. The power of each step should be at least 80%.

Based on achieved information of a study by Galon et al. [20] in CRC patients, the density of tumor infiltrating T cells is a relevant prognostic parameter. Patients with a good prognosis had a mean of x-good = 600 CD8 T cells/mm^2^, the group with a bad prognosis had a mean of x-bad = 370 CD8 T cells/mm^2^. The standard deviation in both groups was 50/mm^2^. Further analysis revealed that patients with x = 300 tumor infiltrating CD8 T-cells/mm^2 ^in tumor tissue had a significantly better prognosis. Given this background, a clinical relevant difference between 100 and 200 CD8 T-cells/mm^2 ^seems plausible. The number of cases should be sufficient for a difference of 150 CD8 T-cells/mm^2^.

For the first step, 32 analyzable patients will be needed which equals eight patients for each of the four groups. The nominal power of ANOVA is around 90%. For the second step (comparison of the best group, Hsu 2006), the power for n = 32 is 95%. Not all of the randomized patients could be analyzed with respect to the primary objective. A failure of 10 - 15% has to be assumed due to poor tissue quality or technical problems. Therefore, 40 patients should be randomized.

The calculated number of cases was carried out with PASS 2005 (Hintze J, 2004): NCSS and PASS. Number Cruncher Statistical Systems. Kaysville, Utah. http://www.ncss.com) with the procedure for multiple comparisons by Jason C. Hsu (1996) (Multiple Comparisons: Theory and methods, Chapman & Hall). The analysis of the primary objective results confirmatory on a multiple level of significance of p < 0.05 by ANOVA based on the calculated numbers of cases. The analysis of the secondary objective results is descriptive by determining the means, the quartiles as location parameter and the standard deviation and range as dispersion parameters. The correlation between the parameters will be quantified by the Pearson or Spearman test. The EORTC QLY-C30 single-item and multi-item subscales will be scored and linearly transformed according to algorithms recommended by the EORTC. Using two-sample t-tests for continuous and Pearsons's X^2 ^Test for categorical variables, the intervention and the control arms will be compared in terms of clinical characteristics at time of randomisation and in terms of baseline QOL scores. Repeated measures ANOVA will be applied to test for changes in the continuous secondary endpoints - e.g. plasma cytokine levels- as a function of treatment arms.

### Follow up

A routine follow up for cancer patients will be done including physical examination of the patients, tumor markers (CEA), CT/MRI, chest X-ray every three months. Disease-free survival and overall survival will be recorded. Notably, the further clinical management and postoperative treatment of the patients will not be influenced by the results of this study. The decision whether patients will receive an adjuvant treatment (e.g. chemotherapy) is left to the discretion of the treating oncologist.

### Data management and quality assurance

The coordination of this trial lies by the Department of Surgery and the Radiation Oncology Department of the German Cancer Research Center (DKFZ). Data regarding T cell detection in blood and bone marrow of all patients will be entered in a password-protected database at the Department of General-, Visceral- and Transplantation Surgery, University of Heidelberg and at the German Cancer Research Center. The clinical data of all included patients will be centrally collected in a database located at the Department of Radiation Oncology at dkfz. The clinical and laboratory data will be merged in the password-protected database at the Department of General-, Visceral- and Transplantation Surgery, University of Heidelberg, Germany and at the DKFZ.

## Discussion

The primary objective of this clinical phase I/II study is to investigate if tumor conformal low dose external beam radiation using 6 MV photons will lead to an increased number of tumor infiltrating T cells at two days after irradiation in resectable liver metastases of colorectal cancer.

The efficient radiotherapeutic treatment of colorectal liver metastasis is still a challenging task. Eradicating radiation doses are only applicable for small colorectal liver metastases because of the high radiation toxicity to the liver [21]. Therefore, the development of new therapeutic strategies is required. Cellular immunotherapy is a promising complimentary tumor treatment principle. Although the ability of tumor rejection of tumor specific T lymphocytes is well documented [22,23], the effectiveness of this approach could not be translated into clinical trials. It appears to be essential, that the tumor context has to be specifically modulated to raise the efficiency for immunotherapy.

Low dose ionizing radiation offers the possibility of a selective induction of a proinflammatory milieu. In contrast to systemic adjuvants, a localized synergistic tumor effect is possible by causing a release of tumor antigens from necrotic tumor cells and depletion of regulatory T cells [24]. Ionizing radiation was initially considered as an immune-suppressive treatment modality [25], but it has also revealed its potential to enhance immunity.

Therefore, the hypothesis for the current study was that an improved T cell infiltration and activation in the tumor as well as a superior systemic T cell immunity against tumor antigens can be induced by low dose radiation therapy. However, the role of radiation as an independent immune-enhancer is still under investigation. While encouraging preclinical data are now available, their translation to the clinic - alone or in combination with other forms of immune therapy - is just beginning.

## Competing interests

The authors declare that they have no competing interests.

## Authors' contributions

CR participated in the study design, trial coordination and conduction, patient recruitment, translational studies, and wrote the manuscript. CT participated in protocol design, ethical committee and BfS applications, patient recruitment and translational studies. HSW participated in developing the protocol concept, in the ethical committee application, trial coordination and patient recruitment. NNR participated in the patient recruitment and trial coordination. MK participated in the patient recruitment and trial coordination. FK carried out the immuno monitoring and participated in trial coordination. FR participates in trial conduction.

JD participates in trial conduction. LE made the statistics. MWB participates in trial conduction. PB participated in developing the study concept, trial conduction and coordinated the immuno monitoring. PEH coordinated the study concept, the trial conduction and translational studies and protocol design, led the approval committee applications. JW participated in developing the study concept, and trial coordination and conduction. All authors read and approved the final manuscript.

## Pre-publication history

The pre-publication history for this paper can be accessed here:

http://www.biomedcentral.com/1471-2407/11/419/prepub
